# Utilizing induced neural stem cell‐based delivery of a cytokine cocktail to enhance chimeric antigen receptor‐modified T‐cell therapy for brain cancer

**DOI:** 10.1002/btm2.10538

**Published:** 2023-05-29

**Authors:** Alex S. Woodell, Elisa Landoni, Alain Valdivia, Andrew Buckley, Edikan A. Ogunnaike, Gianpietro Dotti, Shawn D. Hingtgen

**Affiliations:** ^1^ Division of Pharmacoengineering and Molecular Pharmaceutics, UNC Eshelman School of Pharmacy University of North Carolina at Chapel Hill Chapel Hill North Carolina USA; ^2^ Lineberger Comprehensive Cancer Center University of North Carolina at Chapel Hill Chapel Hill North Carolina USA; ^3^ Center for Nanotechnology in Drug Delivery, UNC Eshelman School of Pharmacy University of North Carolina at Chapel Hill Chapel Hill North Carolina USA; ^4^ Department of Microbiology and Immunology University of North Carolina at Chapel Hill Chapel Hill North Carolina USA

**Keywords:** chimeric antigen receptor T cell, CSPG4 antigen, glioblastoma, IL‐15, neural stem cell, RANTES

## Abstract

Chimeric antigen receptor (CAR)‐modified T‐cell therapy has shown enormous clinical promise against blood cancers, yet efficacy against solid tumors remains a challenge. Here, we investigated the potential of a new combination cell therapy, where tumor‐homing induced neural stem cells (iNSCs) are used to enhance CAR‐T‐cell therapy and achieve efficacious suppression of brain tumors. Using in vitro and in vivo migration assays, we found iNSC‐secreted RANTES/IL‐15 increased CAR‐T‐cell migration sixfold and expansion threefold, resulting in greater antitumor activity in a glioblastoma (GBM) tumor model. Furthermore, multimodal imaging showed iNSC delivery of RANTES/IL‐15 in combination with intravenous administration of CAR‐T cells reduced established orthotopic GBM xenografts 2538‐fold within the first week, followed by durable tumor remission through 60 days post‐treatment. By contrast, CAR‐T‐cell therapy alone only partially controlled tumor growth, with a median survival of only 19 days. Together, these studies demonstrate the potential of combined cell therapy platforms to improve the efficacy of CAR‐T‐cell therapy for brain tumors.

## 1 INTRODUCTION

Cell therapies are reshaping the landscape of cancer treatment strategies. Chimeric antigen receptor (CAR)‐modified T cells have demonstrated robust efficacy and durable responses in patients with hematological malignancies. Strategies that translate these therapeutic benefits to patients with solid tumors is under intense investigation. A well‐studied approach to enhance CAR‐T therapy involves co‐administration of supporting cytokines. However, immunomodulators are known to elicit toxicities when administered systemically,^1^, ^2^ emphasizing the need for targeted delivery. Direct engineering of T cells has been explored, but variability in production levels remain, and the short half‐life of directly conjugated agents may limit the therapeutic window.

In previous studies, we and others have demonstrated that engineered neural stem cells (NSC) are a promising platform for delivering agents.^3^, ^4^, ^5^, ^6^, ^7^ NSCs have a unique tumor‐homing property which enables them to seek out solid and invasive tumor foci.^7^ When engineered to express specific gene products, NSCs provide long‐term drug delivery to malignant tissue resulting in marked modulation of tumor volumes or the tumor microenvironment across a variety of preclinical models.^8^, ^9^ NSCs have been engineered to release different cytokines, including interleukin (IL)‐4 (IL‐4),^10^ IL‐12,^11^ and IL‐23.^12^ These studies have shown that efficient NSC‐based cytokine delivery induced regression of the aggressive brain cancer glioblastoma (GBM). However, tumors eventually escaped and mice succumbed to recurring tumors. As such, durability of antitumor response remains a key issue.

Here, we developed a novel strategy in which we leveraged tumoritropic properties of engineered induced neural stem cells (iNSC) to enhance tumor trafficking and persistence of CAR‐T cells. To this end, we used novel iNSCs transdifferentiated from human skin, a clinically relevant murine model of GBM, and clinically applicable CAR‐T cells. Building on findings from previous virotherapies,^13^ we engineered iNSCs to express the chemoattractant RANTES (Regulated upon Activation, Normal T cell Expressed and Secreted; also known as CCL5) and IL‐15, which promotes T‐cell proliferation and persistence.^14^, ^15^ We found that RANTES/IL‐15‐secreting iNSCs enhanced CAR‐T‐cell migration in co‐culture systems and their combination eliminated tumors in GBM xenograft models.

## 2 RESULTS

### 2.1 Investigating the impact of cell‐based RANTES/IL‐15 delivery on CAR‐T‐cell therapy in GBM

**FIGURE 1:**
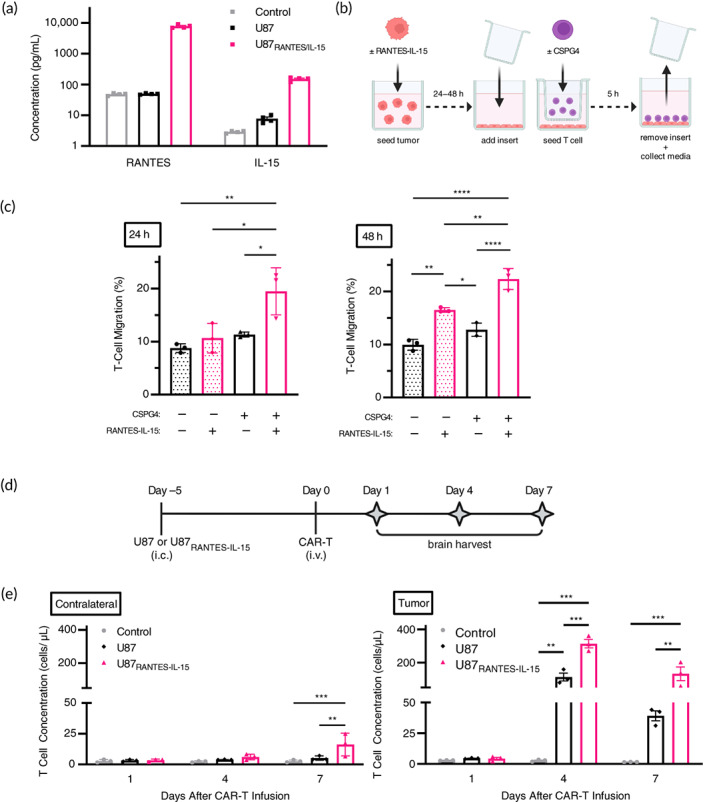
RANTES/IL‐15‐secreting GBM tumor cells enhance T‐cell trafficking and expansion. (a) RANTES and IL‐15 were detected in culture supernatant collected from transduced U87 tumor cells. (b) U87 or U87_RANTES‐IL‐15_ were seeded and cultured for 24 or 48 h. Transwell inserts were added and CSPG4‐CAR‐T or control T cells were seeded therein. T cells were allowed to migrate through inserts for 5 h. Afterward, inserts were removed and media was collected for flow cytometry. (c) Migration of CSPG4‐CAR‐T or control T cells toward U87 or U87_RANTES‐IL‐15_ cultured for 24 or 48 h was evaluated by flow cytometry (*n* = 3). (d) NSG mice were implanted i.c. with U87, U87_RANTES‐IL‐15_, or PBS in the right hemisphere of the brain. Five days later, mice were infused i.v. with CSPG4‐CAR‐T. Mice were sacrificed 1, 4, or 7 days following CSPG4‐CAR‐T infusion. Brains were harvested, bisected along the interhemispheric fissure, and processed for flow cytometry (*n* = 3). (e) CSPG4‐CAR‐T concentrations in single cell suspensions generated from bisected mouse brains (contralateral hemisphere vs tumor hemisphere) were analyzed by flow cytometry. Schematic(s) created with BioRender.com. *p < 0.05, **p < 0.01, ***p < 0.001. CAR‐T, chimeric antigen receptor‐modified T cell; CSPG4‐CAR‐T, CAR‐T cells targeting chondroitin sulfate proteoglycan 4 antigen; GBM, glioblastoma; IL‐15, interuekin 15; NSG, NOD.Cg‐*Prkdc* ^*scid*^ *Il2rg* ^*tm1Wjl*^/SzJ.

To begin investigating cell‐based delivery of RANTES/IL‐15 to enhance CAR‐T‐cell migration and proliferation, we first transduced human U87 GBM cells with a replication‐incompetent retroviral vector (RV) encoding human RANTES and IL‐15 (U87_RANTES‐IL‐15_). Enzyme‐linked immunosorbent assay (ELISA) analysis of conditioned media confirmed cytokine expression, with U87_RANTES‐IL‐15_ cells producing 7721 pg/mL of RANTES and 150 pg/mL of IL‐15 over 72 h (Figure 1a). We then performed cell migration and proliferation assays using CAR‐T cells targeting chondroitin sulfate proteoglycan 4 antigen (CSPG4‐CAR‐T) that we previously identified as a clinically relevant target in GBM.^16^ T‐cell expression of CSPG4‐CAR was confirmed via flow cytometry (Figure S1). Using a transwell assay system, U87 cells were seeded and cultured overnight in growth medium. The next day, T cells were seeded in transwell inserts, allowed to migrate for 5 h, and enumerated via flow cytometry. We found that U87_RANTES‐IL‐15_ increased the migration of control T cells 1.9% at 24 h or 6.6% at 48 h, compared to migration toward control U87 cells (Figure 1b,c). This effect was more pronounced in CSPG4‐CAR‐T cells, with migration increasing 8.8% at 24 h or 9.6% at 48 h.

To investigate the impact of RANTES/IL‐15 on T‐cell migration in vivo, we implanted U87_RANTES‐IL‐15_ or control U87 cells into brains of immunodeficient NOD.Cg‐*Prkdc* ^*scid*^ *Il2rg* ^*tm1Wjl*^/SzJ (NSG) mice, which display severe defects in innate and adaptive immunity. Five days later, CSPG4‐CAR‐T cells were infused intravenously (Figure 1d). One, 4, or 7 days after CSPG4‐CAR‐T‐cell infusion, brains were harvested, bisected along the interhemispheric fissure, and separated into “tumor” and “contralateral” (nontumor) hemispheres. We then utilized flow cytometry to quantify number of T cells present within each hemisphere. Using this approach, we found that RANTES/IL‐15 secretion significantly increased the amount of T cells present within tumors, with T‐cell accumulation threefold higher in U87_RANTES‐IL‐15_ compared to control U87 cells on Day 4 (314 cells/μL, U87_RANTES‐IL‐15_; 113 cells/μL, U87) and over fourfold higher on Day 7 (133 cells/μL, U87_RANTES‐IL‐15_; 39 cells/μL, U87; Figure 1e). In contrast, we detected only slight differences in T‐cell accumulation between the contralateral hemispheres on Day 7 (16 cells/μL, U87_RANTES‐IL‐15_; 5 cells/μL, U87). Taken together, these data demonstrate a proof of concept that RANTES/IL‐15 enhance T‐cell migration in vitro and in vivo. In addition, T‐cell migration into the brain is selective toward the tumor hemisphere with few cells observed in the contralateral hemisphere.

### 2.2 Generation and characterization of iNSCs expressing RANTES/IL‐15

**FIGURE 2:**
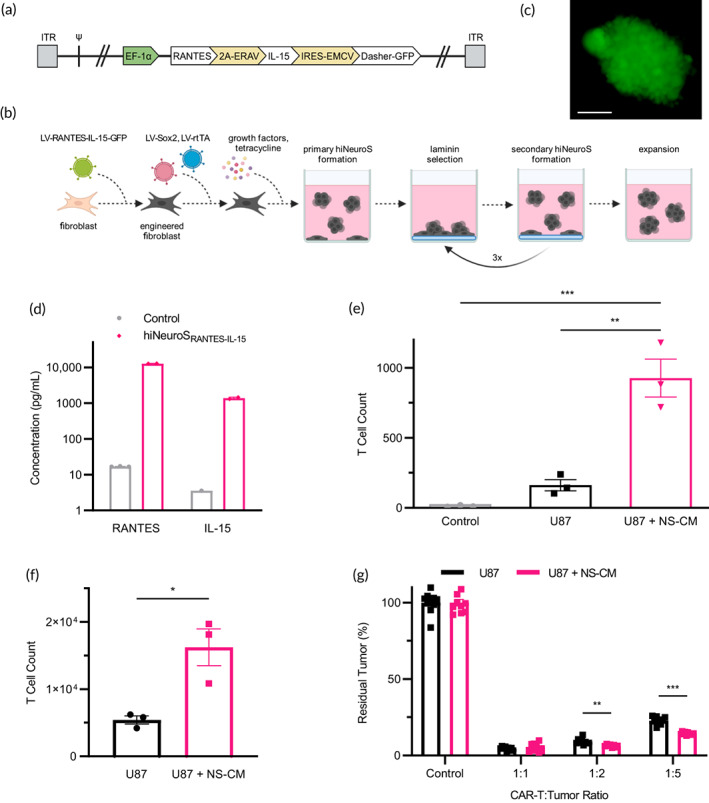
Development and characterization of hiNeuroS_RANTES‐IL‐15_. (a) Schematic representation of lentivirus encoding human RANTES and IL‐15. (b) Human fibroblasts were transduced with LV‐RANTES‐IL‐15‐GFP, LV‐Sox2, and LV‐rtTA in succession. Reprogramming and primary hiNeuroS formation were initiated by switching to NSC growth media supplemented with growth factors and tetracycline. Three rounds of cell cluster selection were performed by serial seeding on laminin‐coated plates. After each round of selection, nonadhering cells were collected and expanded to yield secondary hiNeuroS. (c) Fluorescence imaging of hiNeuroS_RANTES‐IL‐15_ confirms successful LV‐RANTES‐IL‐15‐GFP transduction. Scale bar represents 100 μm. (d) RANTES and IL‐15 were detected in culture supernatant collected from transduced hiNeuroS. (e) Migration of CSPG4‐CAR‐T toward U87 ± NS‐CM or control media using a transwell migration assay was evaluated by flow cytometry (*n* = 3). (f) CSPG4‐CAR‐T counts after 3 days in co‐culture with U87 ± NS‐CM (*n* = 3). (g) Anti‐tumor activity of CSPG4‐CAR‐T after 3 days in co‐culture with U87 ± NS‐CM was assessed by bioluminescence (*n* = 8–10). Schematic(s) created with BioRender.com. *p < 0.05, **p < 0.01, ***p < 0.001. CAR‐T, chimeric antigen receptor‐modified T cell; CSPG4‐CAR‐T, CAR‐T cells targeting chondroitin sulfate proteoglycan 4 antigen; NSC, neural stem cell.

Engineered iNSCs possess great potential for cell therapy applications due to their innate tumor‐homing and ability to provide sustained delivery of biologic agents. To investigate whether these unique properties of iNSCs can enhance anti‐tumor properties of CAR‐T cells, we first constructed a lentiviral vector (LV) encoding human RANTES and IL‐15 (Figure 2a). In this construct, RANTES and IL‐15 were cloned under control of the EF‐1 alpha promoter and separated by a 2A‐peptide sequence. An IRES element driving green fluorescent protein (GFP) expression was also included to quantify transduction efficiency. Using our well‐established methodology for converting human fibroblasts into second‐generation iNSCs, human‐induced neurospheres (hiNeuroS),^17^ we engineered RANTES/IL‐15‐secreting hiNeuroS (hiNeuroS_RANTES‐IL‐15_) (Figure 2b). Robust expression of the GFP reporter gene was detected by fluorescence microscopy (Figure 2c). ELISA analysis of conditioned media confirmed RANTES/IL‐15 secretion, with hiNeuroS_RANTES‐IL‐15_ producing 12,777 pg/mL of RANTES and 1377 pg/mL of IL‐15 over 72 h (Figure 2d).

We used a transwell assay to investigate the effect of hiNeuroS_RANTES‐IL‐15_ on CAR‐T‐cell migration. U87 cells were seeded and cultured overnight in hiNeuroS_RANTES‐IL‐15_ conditioned media (NS‐CM) or control media. The next day, CSPG4‐CAR‐T cells were seeded in transwell inserts and allowed to migrate for 5 h. Flow cytometry was used to enumerate T cells, leading to sixfold greater CAR‐T‐cell migration in NS‐CM compared to control media (927 cells, U87 + NS‐CM; 162 cells, U87). To investigate the effect of iNSC‐delivered RANTES/IL‐15 on T‐cell proliferation, CSPG4‐CAR‐T cells were cultured in NS‐CM or control media for 72 h. Flow cytometry analysis revealed threefold greater CAR‐T expansion in NS‐CM compared to control media (16,219 cells, U87 + NS‐CM; 5411 cells, U87; Figure 2e,f).

To explore the ability of hiNeuroS_RANTES‐IL‐15_ to enhance the anti‐tumor cytotoxicity of CAR‐T therapy, FLuc^+^ U87 cells were co‐cultured with increasing amounts of CSPG4‐CAR‐T cells in the presence of NS‐CM or control media for 72 h. Luciferase assays showed viability of U87 cells in control media was reduced by 96%, 91%, and 77% at CAR‐T: tumor ratios of 1:1, 1:2, and 1:5, respectively (Figure 2g). We detected a further increase in U87 tumor‐killing in the presence of NS‐CM, with viability reduced by 91% and 85% at CAR‐T:tumor ratios of 1:2 and 1:5, respectively. Taken together, these results suggest that hiNeuroS_RANTES‐IL‐15_ can enhance migration, expansion, and tumor‐killing of CAR‐T cells in vitro.

### 2.3 hiNeuroS_RANTES‐IL‐15_ enhances CAR‐T‐cell therapy for GBM

**FIGURE 3:**
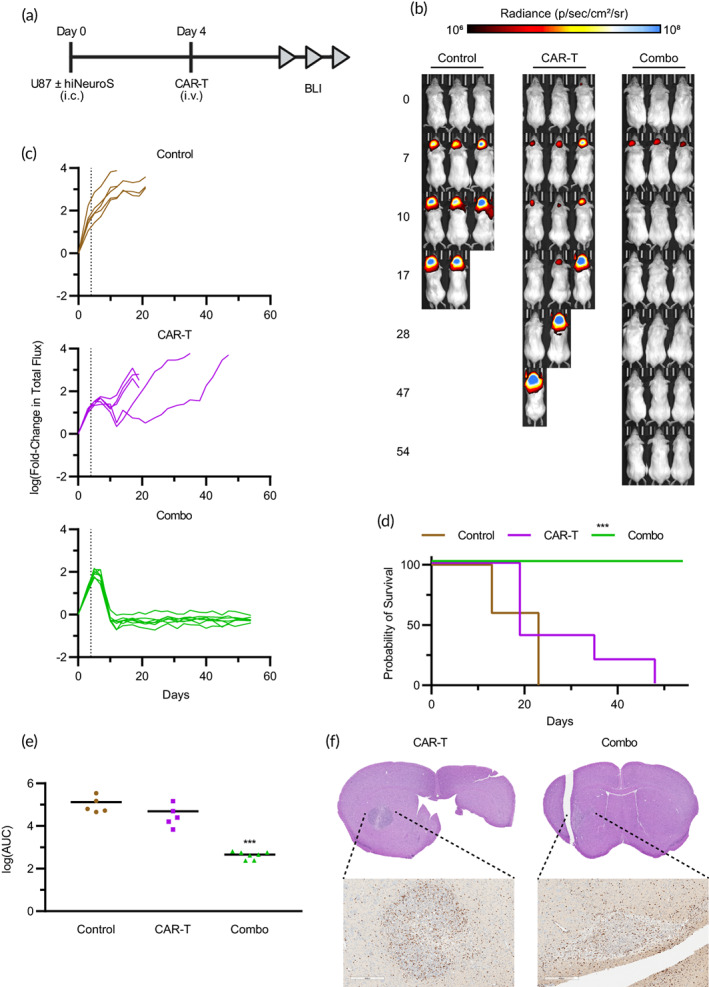
Combination hiNeuroS_RANTES‐IL‐15_ + CAR‐T therapy increases CAR‐T infiltration and eradicates non‐invasive GBM. (a) Schematic representation of study design. NSG mice were implanted i.c. with FLuc^+^ U87 ± hiNeuroS_RANTES‐IL‐15_ in the right hemisphere of the brain. Four days later, mice were infused i.v. with CSPG4‐CAR‐T or PBS (*n* = 5–7). (b) Representative bioluminescent images depict U87 tumor growth. (c) Luminescence was quantified by calculating total flux within a region of interest centered on the head, then normalizing to Day 0 values. Dotted line signifies CAR‐T infusion. (d) Kaplan–Meier survival curves. (e) AUC was calculated for each normalized tumor growth curve. (f) CAR‐T tumor infiltration (brown) was assessed by immunohistochemistry. Schematic(s) created with BioRender.com. ***p < 0.001. CAR‐T, chimeric antigen receptor‐modified T cell; CSPG4‐CAR‐T, CAR‐T cells targeting chondroitin sulfate proteoglycan 4 antigen; GBM, glioblastoma; NSG, NOD.Cg‐*Prkdc* ^*scid*^ *Il2rg* ^*tm1Wjl*^/SzJ.

We next investigated the impact of hiNeuroS_RANTES‐IL‐15_ on CAR‐T‐cell therapy in vivo, using an established human GBM model. FLuc^+^ U87 cells were implanted intracranially with or without hiNeuroS_RANTES‐IL‐15_ in NSG mice (Figure 3a). Serial BLI was then used to monitor tumor growth. Four days later, CSPG4‐CAR‐T cells were infused intravenously while a group of mice remained untreated. We found that CAR‐T cells alone induced a slight inhibition in tumor growth at early time points, reducing GBM volumes 82‐fold by Day 10 (Figure 3b,c). However, the tumors rapidly escaped CAR‐T therapy, increasing in volume 10‐fold by Day 20. The animals succumbed to the large tumor volumes, showing a median survival of just 19 days (Figure 3d) and threefold reduction in area under the curve (AUC) (Figure 3e). In contrast, CAR‐T cells infused in hiNeuroS_RANTES‐IL‐15_‐treated mice markedly inhibited tumor growth, reducing tumor volumes 2538‐fold by Day 10 with most tumors decreasing below the level of detection. The dramatic reduction in tumor volumes led to significant improvements in survival, with all tumor‐bearing mice treated with the combination therapy surviving through 60 days post‐treatment and 290‐fold reduction in AUC.

To confirm that antitumor effects of the combination of CAR‐T cells and hiNeuroS_RANTES‐IL‐15_ were associated with enhanced CAR‐T‐cell migration and persistence, we measured the number of CAR‐T cells within GBM. Following the same treatment regimen as above, a subset of mice were sacrificed 10 days after intravenous CAR‐T‐cell infusion. Brains were collected and immunohistochemistry (IHC) was performed to define the distribution and number of CAR‐T cells within the tumor (Figure 3f). Tumor volumes were larger in mice treated with CAR‐T cells alone, while mostly scar tissue remained following CAR‐T‐cell therapy in hiNeuroS_RANTES‐IL‐15_‐treated mice. Anti‐human CD3 staining showed a high degree of CAR‐T‐cell infiltration into tumors in both treatment groups. Taken together, these findings demonstrate that CAR‐T therapy alone is insufficient to kill GBM in vivo. However, when combined with hiNeuroS_RANTES‐IL‐15_ cells, CAR‐T therapy can completely eradicate tumors as a result of enhanced T‐cell migration and persistence.

## 3 DISCUSSION

NSCs are an emerging new platform for drug delivery in cancer therapy. These cells possess the natural ability to home toward malignant tumors. Furthermore, NSCs can be engineered to express a variety of cytotoxic agents. Although NSCs have been successfully utilized in a wide‐range of preclinical and clinical cancer therapy applications,^8^, ^9^ their potential for co‐opting the immune system has yet to be explored. In this study, we shift away from traditional NSC delivery strategies that rely on secretion of toxic antitumor agents with deleterious off‐target effects in favor of naturally occurring soluble factors that enhance the functionality of immune cells.

RANTES and IL‐15 were previously shown to enhance CAR‐T‐cell migration in a neuroblastoma model.^13^ However, CAR‐T‐cell entry into the CNS is obstructed by unique anatomical barriers that are further complicated by the immunosuppressive properties of GBM. Using U87_RANTES‐IL‐15_, we demonstrated increased CAR‐T‐cell migration into the brain following systemic infusion. CAR‐T infiltration peaked on Day 4, then began to decline by Day 7. This timeline is consistent with previously published data on CAR‐T‐cell tissue biodistribution, which showed peak peripheral tumor infiltration 2 days postinjection and subsequent decline by Day 5.^18^ However, we did not explore the long‐term persistence of CAR‐T cells, which have been detected intracranially for up to 159 days in CNS lymphoma models.^19^ Since CAR‐T‐cell longevity is closely tied to the presence of co‐stimulatory domains, we expect ours could persist for up to 3 months based on the presence of CD28 in our CSPG4‐CAR construct.^20^ In the presence of IL‐15, their longevity may be extended even further as previously evidenced.^21^ Although we did not directly measure in vivo cytokine levels in tumor tissue, we believe these would be comparable to those measured in vitro due to similar cell numbers and incubation times used in these experiments.

**FIGURE 4:**
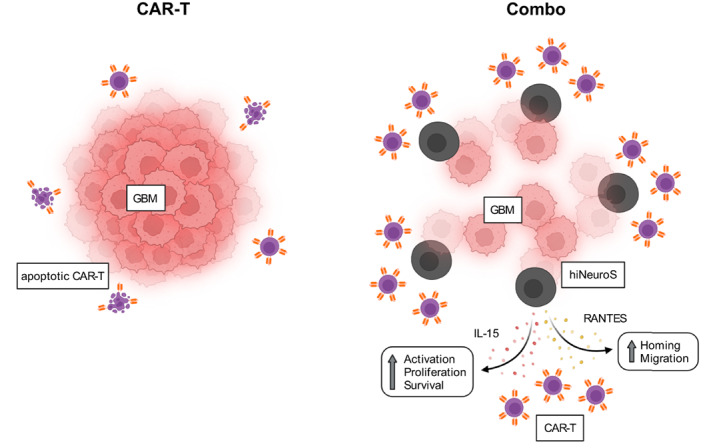
hiNeuroS cytokine delivery platform enhances CAR‐T therapy for GBM. CAR‐T therapy alone results in low lymphocyte infiltration and apoptosis due to the immunosuppressive tumor microenvironment in GBM. When combined with tumor‐homing hiNeuroS_RANTES‐IL‐15_, CAR‐T efficacy is enhanced as a result of increased lymphocyte infiltration, activation, proliferation, and survival. Schematic(s) created with BioRender.com. CAR‐T, chimeric antigen receptor‐modified T cell; GBM, glioblastoma.

Next, we generated a therapeutic iNSC platform, hiNeuroS, for the delivery of RANTES/IL‐15 (Figure 4). Reprogrammed iNSCs offer a distinct clinical advantage over endogenous “off‐the‐shelf” NSCs. They are manufactured from a patient's own cells, thereby reducing the risk for immunogenicity and increasing persistence.^8^, ^9^ Conversion of somatic cells into iNSCs is accomplished via direct and indirect strategies. The former, used to generate hiNeuroS, introduces lineage‐specific transcription factors, while the latter relies on classic Yamanaka factors.^22^ Indirect transdifferentiation has been associated with the formation of teratomas, a type of germ cell tumor, due to passage through a pluripotent intermediate stage.^23^ Direct transdifferentiation, used here, bypasses this intermediate phase, making it a safer cellular conversion strategy.

In addition to the aforementioned safety benefits, hiNeuroS also enjoy a variety of functional enhancements. Previously, we demonstrated that hiNeuroS possess unique transcriptional profiles compared to parent fibroblasts and first‐generation iNSCs, including upregulation of genes related to tumor‐homing migration, stemness, and proliferation.^17^ In vivo, hiNeuroS proliferated for up to 1 week and showed a twofold increase in transhemispheric migration to tumor foci and a threefold increase in persistence compared to traditional iNSCs following intracerebroventricular infusion. These properties make hiNeuroS an ideal vehicle for sustained and targeted cytokine delivery. Here, we used a non‐invasive GBM cell line to assess efficacy of our combination cell therapy. However, it would be interesting to fully explore the therapeutic potential of hiNeuroS against an invasive GBM tumor model in future studies.

A key benefit of using hiNeuroS_RANTES‐IL‐15_ is illustrated in Figure 2g: RANTES/IL‐15 increases tumor killing at low CAR‐T:tumor ratios. Since 2017, the US Food and Drug Administration (FDA) has approved five CAR‐T therapies for the treatment of cancers. The acquisition cost for a single dose of these therapies ranges from $373,000 to $475,000,^24^ placing a substantial financial burden on patients and payers alike. Reducing the number of CAR‐T cells required per dose via supplementation with immune‐modulating iNSCs could significantly reduce these costs. Since there are no FDA‐approved NSC therapies on the market, it is difficult to predict the acquisition cost of a clinical grade product. However, a recent study estimated the manufacturing costs for commercial scale expansion of stem cells would be up to 1000‐fold less than CAR‐T therapy.^25^

In the in vivo efficacy study, we co‐implanted tumor and iNSCs into the brain prior to administering CAR‐T cells intravenously. We chose this design to simplify the technical aspects of the study and demonstrate proof‐of‐concept that a stem cell‐delivered cytokine cocktail enhances CAR‐T penetration and efficacy. In this approach, we saw a clear and substantial benefit from iNSC‐based delivery of RANTES/IL‐15. However, this study failed to address how the combination cell therapy interacts with a pre‐established brain tumor as observed in clinical application. Now that we have established preclinical proof‐of‐concept, we are exploring more complicated studies that mirror different aspects of clinical treatment, including resection/recurrence models, additional supportive cytokines, and different CAR‐T variants.

Another limitation of this study is that we used an immune deficient mouse model to assess in vivo efficacy. Although this allowed us to use clinically relevant human GBM, iNSC, and CAR‐T cells, it also limited our ability to address potential interactions of RANTES/IL‐15 with innate immunity. Previously, IL‐15 was shown to induce production of pro‐inflammatory mediators, tumor necrosis factor alpha (TNF‐α) and IL‐6, in microglia,^26^ while RANTES‐induced astrocytes to release monocyte chemoattractant protein‐1 and TNF‐α.^27^ Since both are implicated in the amplification of inflammatory responses in the CNS, additional studies are necessary to assess the potential for iNSC‐induced neurotoxicity in immune competent animals.

Finally, we cannot exclude the possibility that other factors released by iNSCs may contribute to the beneficial immunomodulatory effects observed in the present study. We previously mapped the transcriptomic profile of hiNeuroS to investigate differences in gene expression associated with stemness, tumor‐homing, and proliferation.^17^ However, additional studies are necessary to explore genes which may be implicated in CAR‐T therapy.

## 4 CONCLUSIONS

In summary, this study demonstrates the first ever use of NSCs as immune modulators. We showed that our next‐generation iNSCs provide a stable source of RANTES and IL‐15 cytokines locally at the tumor site, which enhanced CAR‐T‐cell migration and proliferation. This iNSC‐mediated immune support resulted in complete tumor remission and extended survival indefinitely using a solid GBM tumor model. Together, these data highlight a new application for personalized NSC therapy that can both expand the scope and reduce the cost of clinical CAR‐T therapy.

## 5 MATERIALS AND METHODS

### 5.1 Viral vectors

The following LVs were used in this study and have been described previously: mCherry fused to firefly luciferase (LV‐mCherry‐Fluc), a tetracycline‐inducible Sox2 (LV‐Sox2), and a reverse tetracycline‐controlled transactivator (LV‐rtTA).^28^ A RV encoding RANTES and IL‐15 (RV‐RANTES‐IL‐15) was provided as a gift from G. Dotti (Lineberger Comprehensive Cancer Center). We combined RANTES/IL‐15 coding regions from RV‐RANTES‐IL‐15 with an IRES‐GFP element to generate a RANTES‐IL‐15‐GFP plasmid on a lentiviral backbone (ATUM), which was subsequently packaged as a LV by the Duke University Viral Vector Core.

### 5.2 Cell lines

The U87 glioma cell line and human telomerase reverse transcriptase (hTERT)‐immortalized normal human fibroblasts (NHF1) were obtained from the American Type Culture Collection and as previously described.^29^ Cells were grown in vitro as previously described and tested for mycoplasma.^29^ U87 cells were transduced with LV‐mCherry‐FLuc and/or RV‐RANTES‐IL‐15. We verified that U87 cells retain surface expression of the target neoantigen, CSPG4, using an anti‐human chondroitin sulfate fluorochrome‐conjugated monoclonal antibody (BD Biosciences).

### 5.3 hiNeuroS generation

NHF1 fibroblasts were transduced with LV‐RANTES‐IL‐15‐GFP, followed by a cocktail of LV‐Sox2 and LV‐rtTA as previously described.^29^ Following transduction, hiNeuroS were generated as previously described.^17^ Briefly, cells were cultured in ReNcell NSC maintenance medium (Sigma‐Aldrich) supplemented with epidermal growth factor (epidermal growth factor [EGF]; 20 ng/mL, Gemini Bio‐Products), fibroblast growth factor (fibroblast growth factor [FGF]; 20 ng/mL, Sigma‐Aldrich), and doxycycline (2 μg/mL, Sigma‐Aldrich). EGF, FGF, and doxycycline were replenished every other day. Newly formed spheres were collected and seeded on pre‐coated laminin (10 mg/mL, Sigma‐Aldrich) plates. The next day, nonadherent cells were washed away and new spheres were allowed to regenerate from the remaining attached cells. This process of laminin selection was completed in triplicate. Following laminin selection, hiNeuroS were maintained in low attachment Nunclon Sphera (Thermo Scientific) flasks to promote sphere formation and expansion.

### 5.4 CAR‐T generation

Retroviral supernatants were prepared by transient transfection of 293 T cells and used to transduce T cells.^30^ T cells were transduced with the retroviral vector encoding the CSPG4‐specific scFv (763.74(B)), the CD8a stalk and transmembrane domain, the CD28 intracellular domain and CD3z chain (CSPG4.CAR).^31^ Buffy coats from healthy volunteer blood donors were purchased from the Gulf Coast Regional Blood Center. Peripheral blood mononuclear cells (PBMC) were isolated by Lymphoprep (Accurate Chemical and Scientific Corporation) density‐gradient centrifugation. T cells isolated from PBMCs were cultured in complete T‐cell medium, consisting of 45% Click's medium (Irvine Scientific), 45% RPMI‐1640 (Hyclone), 10% FBS (Hyclone), 1% l‐glutamine (Gibco), and 1% penicillin/streptomycin (Gibco). T cells were activated, transduced, and expanded in complete medium with IL‐7 (10 ng/mL, PeproTech) and IL‐15 (5 ng/mL, PeproTech) as previously reported.^30^

### 5.5 Enzyme‐linked immunosorbent assay

To measure in vitro cytokine production, cells were seeded at 2.5e5 cells/mL in 24‐well plates. Supernatants were collected 72 h later and analyzed for the production of RANTES and IL‐15 using specific ELISA kits (R&D Systems).

### 5.6 Migration assays

The in vitro migration assays were conducted as previously described using 5‐μm pore 24‐well transwell plates (Corning Life Science) and complete T‐cell medium.^32^ To assess in vivo T‐cell migration, we used female NSG mice (Jackson Laboratory). Stereotaxic surgery was performed on mice to engraft 1e5 U87 tumor cells mixed with or without 5e5 hiNeuroS_RANTES‐IL‐15_ intracranially (AP = 0.6 mm, ML = 2.0 mm, DV = 3.0 mm). Five days later, mice were intravenously infused with either control or 1e7 CSPG4‐CAR‐T. We sacrificed mice 1, 3, or 5 days following T‐cell infusion and harvested their brains, which were subsequently bisected along the interhemispheric fissure, dividing them into tumor and contralateral hemispheres. Each hemisphere was processed separately for flow cytometry as previously described.^33^

### 5.7 Flow cytometry

The expression of the 763.74(B) CARs was assessed using specific anti‐idiotypic antibodies (MK2‐23 mAb) kindly provided by S. Ferrone (Massachusetts General Hospital), followed by staining with a secondary rat anti‐mouse antibody (PE, clone X56) from BD Biosciences. T cells were stained with an anti‐human CD3 fluorochrome‐conjugated monoclonal antibody (Biolegend). Dead cells were excluded using a nucleic acid stain, Helix NP NIR (Biolegend). Fluorescence‐activated cell sorting (FACS) data were collected using an LSRFortessa (BD Biosciences) flow cytometer and analyzed with FlowJo (BD Biosciences) software.

### 5.8 Bioluminescent imaging

Serial bioluminescent imaging (BLI) was used to assess tumor growth in vivo as previously described.^29^ Briefly, mice were given d‐luciferin (3 mg/mouse in 200 μL phosphate buffered saline (PBS)) by intraperitoneal injection. Fifteen minutes later, photon emission was measured using an IVIS Spectrum (PerkinElmer) imaging system. For in vivo studies, tumor luminescence was quantified using Living Image (PerkinElmer) software and normalized based on the earliest recorded tumor signal. AUC was calculated from normalized luminescence curves using last observation carried forward for deceased animals. For in vitro studies, d‐luciferin (1:100) was added directly to tissue culture plates and luminescence was measured 10 min later using a Gen5 (Agilent) microplate reader.

### 5.9 Tissue processing

Following anesthetization with 5% isoflurane, mice were perfused via intracardiac puncture with 10 mL PBS and 10% formalin. Brains were harvested, incubated at room temperature in 10% formalin for 72 h, and transferred to 70% ethanol. Mouse brains were trimmed in a coronal orientation through the injection site, then processed and embedded as fixed formalin paraffin blocks. Sections were obtained at 5 μm and mounted onto positively charged slides.

### 5.10 Immunohistochemistry

Chromogenic IHC was performed on paraffin‐embedded brain samples. IHC was carried out using the Bond‐III Autostainer (Leica Biosystems). Slides were dewaxed in Bond Dewax Solution (Leica Biosystems) and hydrated in Bond Wash Solution (Leica Biosystems). Heat‐induced antigen retrieval was performed for 20 min at 100°C using Bond‐Epitope Retrieval Solution 1 pH 6.0 (Leica Biosystems). This was followed by a 5‐min peroxide blocking step using Bond Polymer Refine Detection (Leica Biosystems). After pretreatment, slides were incubated for 1 h with 1:500 anti‐human CD3 antibody (ab5690, Abcam). Antibody detection with 3,3′‐diaminobenzidine was accomplished using the Bond Intense R Detection System (Leica Biosystems) supplemented with Novolink Polymer (Leica Biosystems) secondaries. Stained slides were dehydrated and coverslipped with Cytoseal 60 (Thermo Fisher Scientific). Positive controls were included for each assay. IHC‐stained slides were digitally imaged in the Aperio AT2 (Leica Biosystems) using a ×20 objective.

### 5.11 Co‐culture experiments

Tumor cells were seeded in tissue culture plates (1e4 cells/well in 96‐well plates for cytotoxicity assay and 1e5 cells/well in 24‐well plates for proliferation assay) and cultured overnight. CSPG4‐CAR‐T cells (1e4, 5e3, or 2e3 cells/well for cytotoxicity assay and 5e4 cells/well for proliferation assay) were suspended in complete T‐cell medium or hiNeuroS_RANTES‐IL‐15_ conditioned media (supernatant from 2.5e5 cells/mL after 72 h); cell suspensions were added and cultured for an additional 3 days. Residual FLuc^+^ U87 cells were assessed via BLI, while residual T cells were counted based on CD3 expression.

### 5.12 GBM xenograft model

To assess antitumor effects and infiltration of CSPG4‐CAR‐T, we used 23 immunocompromised female NSG mice (Jackson Laboratory). Stereotaxic injections were performed in the right hemisphere of each mouse brain to engraft 1e5 FLuc^+^ U87 or 2.5e5 GBM8 tumor cells mixed with or without 5e5 hiNeuroS_RANTES‐IL‐15_ intracranially (AP = 0.6 mm, ML = 2.0 mm, DV = 3.0 mm). Mice were intravenously infused with 3e6 CSPG4‐CAR‐T or PBS 4 days later. Tumor growth was assessed 3 days per week via BLI.

### 5.13 Statistical analysis

All animal experiment methodology followed the ARRIVE guidelines. Minimization randomization strategy was applied to allocate animals to treatment groups based on bioluminescence values to ensure balance among groups.^34^, ^35^ Data were analyzed using Prism (GraphPad) software. Student's *t*‐test was used when comparing only two groups. One‐way analysis of variance (ANOVA) was used when comparing multiple groups and Kruskal–Wallis test was used for longitudinal studies. Statistically significant ANOVAs and Kruskal–Wallace tests were followed by Bonferroni's and Dunn's multiple comparisons test, respectively. Survival analysis was performed using the Mantel–Cox test. Unless otherwise specified, all values are expressed as mean ± standard error of the mean. An alpha level of 0.05 was used to determine statistical significance.

## AUTHOR CONTRIBUTIONS

**Alex S. Woodell:** Conceptualization (lead); data curation (lead); formal analysis (lead); investigation (lead); methodology (lead); project administration (lead); visualization (lead); writing—original draft (lead); writing—review and editing (lead). **Elisa Landoni:** Conceptualization (supporting); formal analysis (supporting); investigation (supporting); methodology (supporting); validation (supporting); visualization (supporting); writing—review and editing (supporting). **Alain Valdivia:** Conceptualization (supporting); investigation (supporting); methodology (supporting); writing—review and editing (supporting). **Andrew Buckley:** Methodology (supporting); validation (supporting); writing—review and editing (supporting). **Edikan Ogunnaike:** Investigation (supporting); validation (supporting). **Gianpietro Dotti:** Conceptualization (supporting); funding acquisition (supporting); project administration (supporting); resources (supporting); supervision (supporting); writing—review and editing (supporting). **Shawn D. Hingtgen:** Conceptualization (supporting); funding acquisition (lead); project administration (equal); resources (lead); supervision (lead); writing—review and editing (supporting).

## CONFLICT OF INTEREST STATEMENT

Shawn D. Hingtgen has an ownership interest in Falcon Therapeutics, Inc., which has licensed aspects of iNSC technology from UNC‐CH. All remaining authors declare that the research was conducted in the absence of any commercial or financial relationships that could be construed as a potential conflict of interest.

### PEER REVIEW

The peer review history for this article is available at https://www.webofscience.com/api/gateway/wos/peer-review/10.1002/btm2.10538.

## ETHICS STATEMENT

The animals used in this work were housed in sterile animal rooms of an accredited AAALAC laboratory animal facility at UNC‐CH and subjected to procedures described in the correspondent animal protocol (20‐272‐C) approved by its Institutional Animal Care and Use Committee.

## Supporting information


**Figure S1.** CSPG4‐CAR expression in T cells. Following retroviral transduction, the number of CAR^+^ CSPG4‐CAR‐T cells or nontransduced control T cells was determined by flow cytometry (*n* = 5).

## Data Availability

The data that support the findings of this study are available from the corresponding author upon reasonable request.
