# Supplementary figures and images for: Utilizing induced neural stem cell‐based delivery of a cytokine cocktail to enhance chimeric antigen receptor‐modified T‐cell therapy for brain cancer
Source: Bioeng Transl Med. 2023 May 29;8(6):e10538. doi: 10.1002/btm2.10538 (PMC10658508; doi:10.1002/btm2.10538)

Control CSPG4-CAR-T

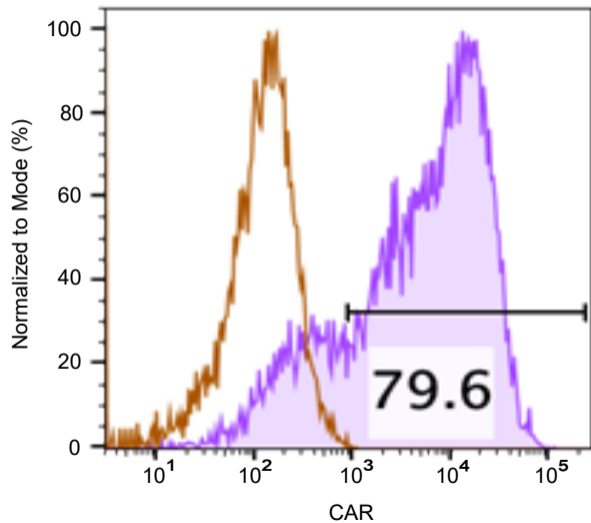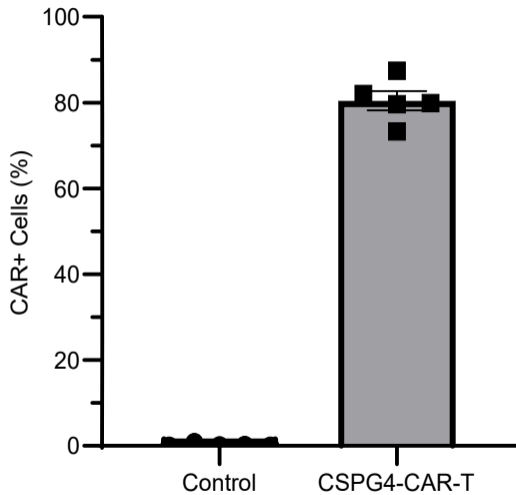

Supplement: Supplementary file 1 — Figure S1. CSPG4‐CAR expression in T cells. Following retroviral transduction, the number of CAR+ CSPG4‐CAR‐T cells or nontransduced control T cells was determined by flow cytometry (n = 5). [file BTM2-8-e10538-s001.pdf]
